# Efficacy of Phytotherapy for Cancer-Related Fatigue: A Systematic Review and Meta-Analysis of Randomized Controlled Trials

**DOI:** 10.3390/diseases14020039

**Published:** 2026-01-26

**Authors:** Silvio Matsas, Ursula Medeiros Araujo de Matos, Carolina Molina Llata, Auro del Giglio

**Affiliations:** 1 Centro de Estudos e Pesquisas de Hematologia e Oncologia (CEPHO), Santo André 09060-650, SP, Brazil; 2 Department of Internal Medicine, University of Connecticut, Farmington, CT 06269, USA; 3 Department of Internal Medicine, Good Samaritan Hospital, Cincinnati, OH 45220, USA

**Keywords:** cancer fatigue, herbal medicine, pooled results

## Abstract

Background: Cancer-related fatigue (CRF) is one of the most common and burdensome symptoms faced by patients with cancer, yet effective drug-based treatments remain limited. In recent years, phytotherapeutic agents have drawn attention as complementary options, supported by plausible anti-inflammatory, antioxidant, and immunomodulatory mechanisms. Methods: We performed a systematic review and meta-analysis to quantitatively synthesize randomized controlled trial evidence on the efficacy of phytotherapeutic interventions for cancer-related fatigue and to assess the certainty of evidence. Databases were searched from inception, with the final search update completed in October 2025. Eligible studies included adults with CRF and compared herbal interventions with placebo controls. Standardized mean differences (SMDs) were pooled using a DerSimonian–Laird random-effects model. We also evaluated risk of bias (RoB 2), publication bias, and certainty of evidence using GRADE. This systematic review and meta-analysis was conducted in accordance with the PRISMA 2020 guidelines. Results: Fourteen trials were included, studying agents such as *Paullinia cupana*, *Panax ginseng*, multi-herbal Traditional Chinese Medicine formulations, and other botanical extracts. Overall, phytotherapy provided a modest improvement in CRF (SMD = 0.31; 95% CI, 0.08–0.53; *p* = 0.022), though heterogeneity was substantial (I^2^ = 56.7%). In subgroup analyses, only the group of “other formulations” demonstrated significant benefit; ginseng and guaraná did not demonstrate statistically significant effects. Most trials had high or unclear risk of bias, and the certainty of evidence was rated very low. Conclusions: Current evidence does not firmly support phytotherapeutic agents as effective treatments for CRF, hindered largely by methodological weaknesses, heterogeneous interventions, and imprecise effect estimates. Even so, the biological rationale and the variability in clinical responses point toward an opportunity for the emerging field of precision herbal oncology. Well-designed, multicenter trials are essential to determine whether phytotherapy can meaningfully contribute to CRF management.

## 1. Introduction

Cancer-related fatigue (CRF) is one of the most common and distressing symptoms experienced by patients with cancer, impacting up to 90% of individuals during active treatment and persisting in a substantial proportion of survivors [1]. CRF is highly prevalent in cancer populations, with some data indicating prevalences of 45.5% and 56.8% in Europe and North America, respectively [2]. Unlike ordinary tiredness, CRF is characterized by a pervasive sense of exhaustion that is disproportionate to activity and not relieved by rest [1]. Its multifactorial pathogenesis encompasses inflammatory activation, neuroendocrine dysregulation, metabolic alterations, mitochondrial dysfunction, sleep disturbance, anemia, and psychological distress [1,3,4].

Pharmacologic management of CRF remains challenging. Agents such as psychostimulants, corticosteroids, and erythropoiesis-stimulating agents provide limited benefit and may result in adverse effects [1,3]. Consequently, interest has grown in complementary and integrative approaches, including phytotherapeutic agents derived from medicinal plants such as *Panax ginseng* (C.A. Mey.), *Paullinia cupana* (Kunth), *Uncaria tomentosa* (Willd. ex Schult.) DC., and *Zingiber zerumbet* (L.) Roscoe ex Sm. [5,6]. Preclinical evidence suggests antioxidant, anti-inflammatory, and immunomodulatory effects through multiple mechanisms of action such as, respectively, direct free-radical scavenging, suppression of pro-inflammatory cytokines, and liver xenobiotic responses [7,8,9,10]. Taken together, these pieces of evidence may plausibly explain the target mechanisms underlying CRF.

**Figure 1:**
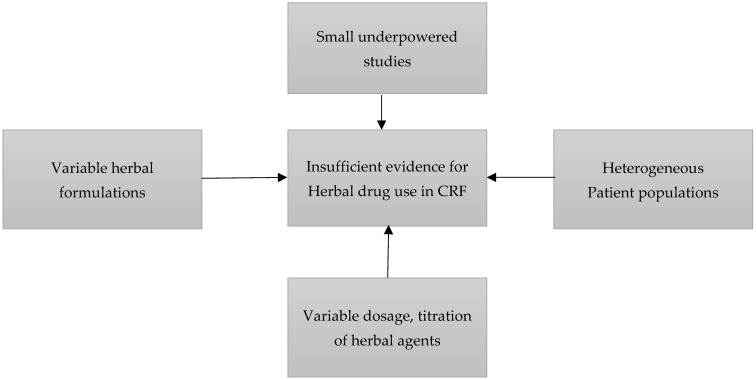
Sources of heterogeneity and evidence uncertainty in herbal CRF trials.

Despite the biological plausibility of phytotherapeutic agents, their clinical role in the management of cancer-related fatigue remains uncertain. Most published trials are small, single-center studies with heterogeneous patient populations, variable herbal formulations, inconsistent dosing regimens, and differing fatigue assessment instruments. Moreover, the lack of standardized phytochemical characterization and limited methodological rigor across studies have contributed to inconsistent findings and imprecise effect estimates (a visual representation of the components contributing to insufficient evidence for herbal drug use in CRF can be seen in Figure 1). As a result, the available literature does not currently allow firm conclusions regarding the efficacy of phytotherapy for CRF, underscoring the need for a rigorous quantitative synthesis and critical appraisal of existing randomized evidence.

Therefore, a pooled study of published randomized controlled trials (RCTs) is needed. The primary objectives of this systematic review and meta-analysis were to quantitatively synthesize data from available RCTs, assess their methodological quality, and ultimately determine the strength of evidence concerning the efficacy of phytotherapy for CRF.

## 2. Methodology

### 2.1. Protocol and Registration

This systematic review and meta-analysis was conducted in accordance with the Preferred Reporting Items for Systematic Reviews and Meta-Analyses (PRISMA) guidelines [11]. The study protocol was registered prospectively in the International Prospective Register of Systematic Reviews (PROSPERO) under registration number CRD420251235154. Study identification, screening, eligibility assessment, and inclusion were performed following PRISMA guidance, and the selection process is summarized using a PRISMA 2020 flow diagram. Data extraction, risk-of-bias assessment, and synthesis methods were implemented in line with PRISMA recommendations for transparency and reproducibility. The PRISMA checklist can be found in the Supplementary Materials.

### 2.2. Eligibility Criteria

Population, Intervention, Comparison, Outcomes, Study design (PICOS) criteria was adopted as follows:

(a) Population (P)

Adult patients (≥18 years) with cancer-related fatigue, regardless of cancer type, stage, or treatment status.

(b) Intervention (I)

Any herbal intervention administered for fatigue management, including but not limited to the following:

- Ginseng (*Panax ginseng*, American ginseng);
- Guaraná (*Paullinia cupana*);
- Traditional Chinese Medicine formulations;
- Other plant-derived interventions.

(c) Comparison (C)

Placebo or standard of care control groups. Active comparator studies were excluded to maintain homogeneity.

(d) Outcomes (O)

Primary outcome: Fatigue severity measured by validated scales (BFI, FACIT-F, MFI, etc.)

Secondary outcomes: Quality of life measures, adverse events, dropout rates

(e) Study Design (S)

Randomized controlled trials (RCTs) published in peer-reviewed journals

### 2.3. Exclusion Criteria

(a) Studies without use of systemic phytotherapeutic agents;
(b) Studies comparing a not-placebo group to phytotherapy;
(c) Studies using a design other than RCT;
(d) Studies written in other language than English.

### 2.4. Information Sources and Search Strategy

(a) Electronic Databases

The following databases were systematically searched from inception, with the final search update completed in October 2025: PubMed/MEDLINE, Embase, Cochrane Central Register of Controlled Trials (CENTRAL), and Google Scholar. We also consulted reference lists of included studies and relevant reviews for additional references.

(b) Search Strategy

Sample PubMed Search Strategy:

(“Fatigue”[Mesh] OR “Cancer Fatigue”[Mesh] OR fatigue[tiab] OR tiredness[tiab]) AND (“Neoplasms”[Mesh] OR cancer[tiab] OR oncology[tiab]) AND (“Plant Extracts”[Mesh] OR “Drugs, Chinese Herbal”[Mesh] OR herb*[tiab] OR ginseng[tiab] OR guarana[tiab] OR “traditional Chinese medicine”[tiab]) AND (randomized controlled trial[pt] OR randomized[tiab] OR placebo[tiab] OR randomly[tiab])

### 2.5. Study Selection Process

Two independent reviewers screened titles and abstracts using Rayyan software [12]. Prior to screening, eligibility criteria were predefined and piloted to ensure consistency in study selection. Each reviewer conducted the screening process blinded to the other’s decisions, and articles clearly not meeting inclusion criteria were excluded at this stage. Full-text articles of potentially eligible studies were then retrieved and assessed independently by the same reviewers to determine final eligibility. Discrepancies at either screening stage were resolved through discussion and consensus; when agreement could not be reached, a third reviewer adjudicated the decision.

### 2.6. Data Extraction and Management

Data were extracted independently by two reviewers using a standardized electronic data collection form developed in Microsoft Excel. The extraction form was piloted on a subset of included studies to ensure clarity, completeness, and consistency across reviewers. Extracted variables included study characteristics (design, setting, sample size), patient demographics, cancer type and treatment phase, details of the phytotherapeutic intervention, fatigue assessment instruments, outcome measures, and safety data. Following independent extraction, the datasets were cross-checked for accuracy and completeness. Any discrepancies between reviewers were resolved through discussion and consensus, with involvement of a third reviewer when necessary. When required, study authors were consulted for clarification of missing or unclear information.

### 2.7. Data Synthesis and Statistical Methods

Effect Measures, quality of evidence, and publication bias assessments:

For continuous outcomes, the Standardized Mean Difference (SMD) with 95% confidence intervals was calculated using Hedges’ g to account for small sample sizes [13]. The DerSimonian–Laird random-effects model was employed due to anticipated clinical and methodological heterogeneity among studies [14]. We assessed publication bias through Funnel plot analysis and we tested funnel plot asymmetry with Egger’s regression test. We conducted a Risk of Bias assessment using the Cochrane Risk of Bias 2 (RoB 2) tool [15].

We employed the R system for statistical computing (version 4.4.2; R Core Team, Vienna, Austria), using random effects model *p* value and heterogeneity calculations.

### 2.8. Ethical Considerations

As this study involved analysis of previously published aggregated data, no individual patient information was accessed or collected. Consequently, the study did not require approval from an institutional Ethics Committee. In addition, all included studies had obtained appropriate ethical approval and informed consent, as reported in their original publications.

## 3. Results

### 3.1. Study Selection

A total of 49 records were identified through database searches, with an additional 2 records identified through citation searching. After removal of 18 duplicates, 33 unique records proceeded to screening. All records were screened at the title and abstract level, and 33 reports were sought for full-text retrieval. Full texts were successfully obtained for 31 records, of which 17 were excluded for clearly predefined reasons: protocols (*n* = 5), absence of a placebo or control group (*n* = 5), non-randomized design (*n* = 3), wrong study design (*n* = 2), wrong population (*n* = 2), topical rather than systemic herbal therapy (*n* = 1), and foreign-language publication not translatable (*n* = 1).

**Figure 2:**
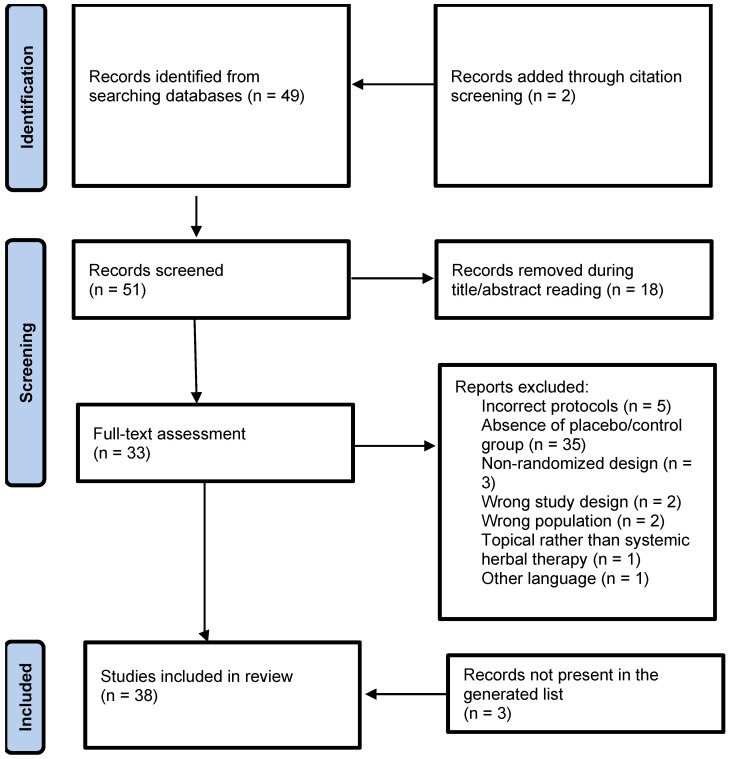
PRISMA diagram of the selection of studies for this meta-analysis.

Ultimately, 14 randomized controlled trials met eligibility criteria and were included in the review and meta-analysis [16,17,18,19,20,21,22,23,24,25,26,27,28,29]. The PRISMA 2020 flow diagram summarizing the study selection process is shown in Figure 2.

### 3.2. Characteristics of Included Studies

The 14 included trials (Table 1) evaluated a range of phytotherapeutic agents—most commonly guaraná, ginseng, ginger, and multi-herbal formulations—administered to patients with cancer-related fatigue (CRF) across various cancer types and treatment phases. Fatigue severity was measured using validated patient-reported instruments including FACIT-F, BFI, MFSI-SF, and SF-36 Vitality. The majority of trials were parallel-group, placebo-controlled RCTs, with sample sizes ranging from 34 to 310 participants.

### 3.3. Risk of Bias Assessment

Risk-of-bias assessment using the Cochrane RoB 2 tool revealed substantial methodological concerns. A total of 14 randomized controlled trials were evaluated using the Cochrane RoB 2 tool. Two studies were judged at low risk of bias, five showed some concerns, and six were classified as high risk. The primary domains contributing to downgraded judgments included issues in randomization procedures, lack of blinding, deviations from intended interventions, and risk of measurement bias in patient-reported fatigue outcomes. High-risk studies frequently involved open-label designs or unclear allocation concealment (Table 2).

### 3.4. Meta-Analysis of the Effect of Phytotherapy on Cancer-Related Fatigue

(a) Overall Effect

**Figure 3:**
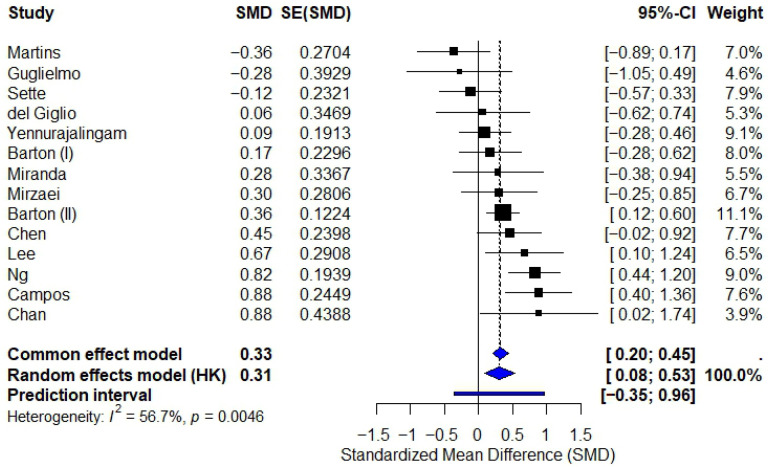
Forest plot of 14 randomized controlled trials evaluating phytotherapy for cancer-related fatigue. Points represent standardized mean differences (SMDs) with 95% confidence intervals for individual trials. The pooled effect (red square) was estimated using a DerSimonian–Laird random-effects model (SMD = 0.31; 95% CI 0.08 to 0.53), indicating a statistically significant benefit of phytotherapy *p* = 0.0043. Heterogeneity was substantial (I^2^ = 56.7%).

Across all 14 trials, the random-effects model demonstrated a significant effect of phytotherapy on CRF: Standardized Mean Difference (SMD): 0.31 (95% CI: 0.08 to 0.53; *p* = 0.0046; Heterogeneity: I^2^ = 56.7%). The corresponding forest plot is shown in Figure 3.

This finding indicates substantial inconsistency across trials, with individual effects ranging from large improvements to moderate worsening of fatigue.

The two most commonly studied herbal subgroups did not demonstrate a statistically significant or clinically meaningful benefit (Table 3). A more general subgroup—named as “Other herbal formulations”—which included diverse formulations had a statistically significant benefit.

### 3.5. Publication Bias

#### Funnel Plot and Statistical Tests

**Figure 4:**
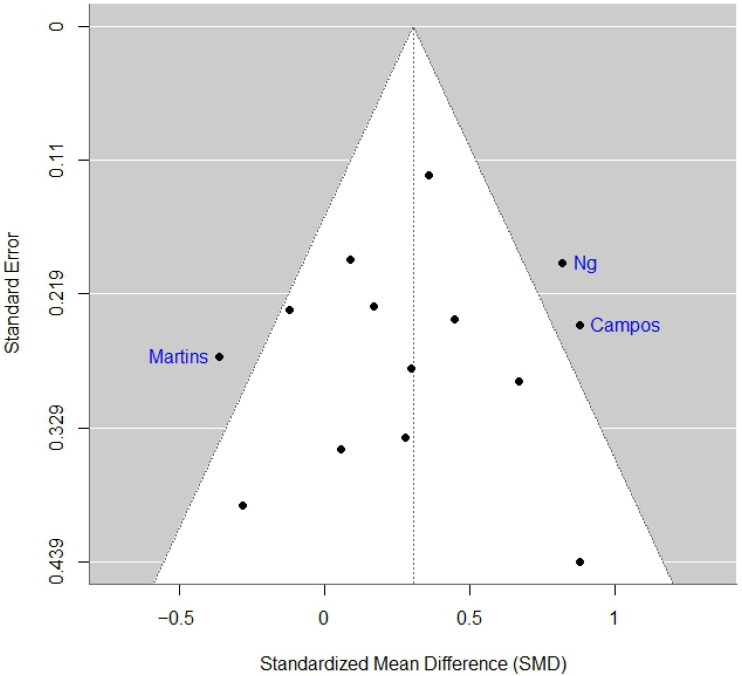
Funnel plot assessing publication bias in 14 randomized controlled trials evaluating phytotherapy for cancer-related fatigue. Each point represents an individual study plotted by standardized mean difference (SMD) on the horizontal axis and Standard Error on the vertical axis. Egger’s regression test did not detect statistically significant small-study effects (intercept = 0.41, *p* = 0.75), suggesting no strong evidence of publication bias, although interpretation should consider the substantial heterogeneity across trials.

Visual inspection of the funnel plot (Figure 4) revealed moderate asymmetry, suggesting missing small-scale negative studies. Egger’s regression test showed no evidence of small-study effects (intercept = 0.41; 95% CI, −0.30 to 1.13; z = −0.31; *p* = 0.75), suggesting no strong evidence of publication bias, although interpretation should consider the substantial heterogeneity across trials.

### 3.6. Certainty of Evidence (GRADE)

Using the GRADE framework, the overall certainty of evidence for the effect of phytotherapy on cancer-related fatigue was rated as very low. Although all included studies were randomized controlled trials (RCTs), the evidence was downgraded across several domains. Risk of bias was a major concern, as only two trials were judged at low risk, while the remaining studies showed either some concerns or high risk—particularly regarding randomization procedures, blinding, and outcome measurement. Inconsistency was substantial (I^2^ = 56.7%), reflecting wide variation in effect estimates and non-overlapping confidence intervals that could not be fully explained by clinical or methodological differences. The overall results, however, point toward a significant improvement of CRF (SMD = 0.31; 95% CI 0.08 to 0.53). Although a significant Egger test (*p* = 0.75) was generated, suggesting low risk of publication bias, this type of bias cannot be excluded due to heterogeneity and the presence of small single-center studies; asymmetry remained plausible due to the predominance of small single-center trials. Subgroup analysis by herbal formulation had no significant results in all three subgroups. Taken together, these limitations resulted in a very low certainty rating, indicating that the true effect of phytotherapy on fatigue may be substantially different from the observed overall estimate.

## 4. Discussion

Use of herbal drugs and integrative approaches in cancer care has increased over time in various regions of the world, with herbal medicine representing the most frequently used form of complementary and alternative medicine among patients with cancer [30,31,32]. A large systematic review and meta-analysis including more than 800,000 patients from 44 countries estimated a global pooled prevalence of herbal medicine use of approximately 22%, with substantially higher rates reported in Africa (≈40%) and Asia (≈28%), and in low- and middle-income countries compared with high-income settings [30]. This underscores the need for structured investigations on the efficacy of these interventions.

This systematic review and meta-analysis synthesizes the most up-to-date evidence on phytotherapeutic interventions for cancer-related fatigue (CRF), incorporating 14 RCTs. The updated pooled analysis demonstrated a statistically significant effect favoring phytotherapy, although heterogeneity was substantial. Ginseng and *Paullinia cupana* were the most commonly investigated herbal drugs. Their subgroup analyses did not reveal statistically significant CRF benefit. The subgroup analysis for a composite of diverse other herbal formulations revealed a significant benefit. Taken together, these results cast significant discrepancies with the overall results and warrant careful interpretation.

Several factors likely contributed to the absence of statistically robust effects on individual herbal classes. First, many included trials were modest in sample size, reducing precision and increasing susceptibility to random variation. Second, methodological limitations were common, including unclear or high-risk randomization procedures, inadequate blinding, and issues related to outcome assessment. Third, heterogeneity in herbal composition, preparation, phytochemical standardization, dosing schedules, and patient populations made it challenging to compare results across studies. These issues reflect broader challenges in botanical research, where variability in extraction methods and phytochemical content may obscure true therapeutic effects [33].

Further challenges stem from limited funding availability for phytotherapy research [34]. Because many botanical agents cannot be patented in their natural or crude extract forms, traditional pharmaceutical funding mechanisms are less accessible [35]. This limitation results in underpowered single-center studies, inconsistent methodological quality, and limited long-term safety follow-up. These structural barriers restrict the development of robust clinical evidence and slow progress in understanding potential mechanisms of action, toxicity profiles, and interactions between phytotherapeutic agents and standard anticancer treatments.

Despite these limitations, phytotherapy remains biologically plausible. Many botanical compounds exhibit anti-inflammatory, antioxidant, immunomodulatory, neuroprotective, and mitochondrial-regulating effects, all of which align with proposed mechanisms underlying CRF pathophysiology [36,37,38,39,40]. Given CRF’s multifactorial nature, therapies composed of diverse bioactive phytochemicals may theoretically offer therapeutic advantages. However, biological plausibility does not substitute for high-certainty clinical evidence, and at present, results remain preliminary.

Our findings are also in opposition to some of the already published data. A meta-analysis with different inclusion criteria investigated the use of Guaraná for treatment CRF and found a significant benefit with the use of this component [41]. This underscores the significant imprecision of phytotherapeutic cancer studies evaluating CRF. Recent ASCO and ESMO studies were also partially discrepant on the use of herbal formulations for CRF [42,43]. While ASCO guideline states that American ginseng may be considered for patients with CRF > 4 weeks during active treatment, some ESMO panel members discourage any use of herbal formulations.

From a broader supportive oncology perspective, phytotherapy and rehabilitation-based interventions have been explored not only for cancer-related fatigue but also for other prevalent symptoms that substantially affect quality of life, including constipation, pain and sleep disturbance [44,45,46]. Integrative approaches combining lifestyle modification, physical rehabilitation, and selected phytotherapeutic agents have shown potential benefits across multiple symptom domains, particularly in supportive and survivorship settings, as highlighted in the recent supportive care literature [47]. Within this context, the findings of the present meta-analysis—characterized by substantial heterogeneity and variable treatment effects—suggest that a uniform, non-stratified application of phytotherapy for fatigue is unlikely to be optimal. Rather than undermining future research, the observed variability in outcomes may reflect underlying differences in patient characteristics, disease stage, treatment context, and phytochemical composition, thereby supporting the rationale for future stratified or precision-guided approaches. Current ASCO and ESMO guidelines do not recommend phytotherapeutic agents for cancer-related fatigue, prioritizing non-pharmacologic interventions due to insufficient high-quality evidence [42,43]. This position is consistent with the low certainty and substantial heterogeneity observed in the present analysis.

While most of the included studies had methodological limitations and a moderate or high risk of bias, a small number of high-quality randomized trials illustrate feasible methodological standards. Notably, the phase III study by Barton et al. [28] employed rigorous randomization, double-blind placebo control, and an almost standardized ginseng dosing. Similarly, the trial by Yennurajalingam et al. [16] demonstrated strong internal validity through careful patient selection, robust blinding, and comprehensive safety reporting. These studies highlight that well-designed phytotherapy trials in CRF are achievable and provide a framework to guide future research.

Our study has a number of limitations. The main limitation of this study is the inclusion of trials encompassing heterogeneous cancer types, diverse herbal formulations with varying compositions, dosages, and titration schedules, and interventions administered at different disease timepoints. This clinical and methodological heterogeneity substantially limits the ability to draw definitive conclusions regarding efficacy. Another limitation is wide subgroup confidence intervals, underscoring small subgroup sizes and imprecision. Therefore, the overall results should be interpreted carefully.

## 5. Conclusions

In conclusion, the current body of randomized evidence does not conclusively support phytotherapeutic agents for reducing CRF. The certainty of evidence is very low, driven by methodological limitations, substantial heterogeneity, and imprecision in effect estimates. This underscores the need for well-designed, adequately powered, multicenter RCTs using standardized herbal preparations. Such studies are essential to clarify the therapeutic potential of phytotherapy for CRF and determine whether specific agents or patient subgroups may truly benefit. Moreover, the perspective for future research include the compared analysis of original scientific publications. Such efforts will be crucial to translating the growing body of observational evidence into clinically actionable, evidence-based phytotherapeutic oncology recommendations.

## Figures and Tables

**Table 1:** Characteristics of the included randomized controlled trials (*n* = 14).

Study	Year of Publication	Years of Enrollment	Years of Implementation	Follow-Up Duration	Population (Inclusion Criteria)	Exclusion Criteria	Study Design	Endpoints	Cancer Types	Performance Status	Fatigue Endpoint	Medication	Key Findings	Country
Guglielmo et al. [23]	2024	2018–2022	2020–2021	12 months (median)	Adults ≥ 18 y with stage II–III breast cancer receiving NAC	Prior systemic therapy; active infection; ECOG > 2	Multicenter retrospective cohort	Primary: pCR; Secondary: DFS, OS, fatigue score change	Breast	ECOG 0–2	Change in FACIT-F from baseline to post-NAC	American ginseng (Panax quinquefolius, Euquinax^®^) 1000 mg/day orally (2 × 500 mg capsules) for 8 weeks; placebo: 2 capsules/day for 8 weeks	While pertuzumab significantly increased pCR rates (absolute gain ~15–20%), fatigue improvements were modest and primarily driven by baseline-to-follow-up changes, with limited evidence of a clinically relevant incremental effect	USA
Lee et al. [22]	2021	May 2018–June 2020	2018–2020	6 weeks total (3-week treatment; assessments through week 6)	Adults > 19 y with confirmed malignant solid tumor; ≥1 month since last major cancer treatment; moderate to severe fatigue (BFI ≥ 4) lasting > 1 month, started or worsened with cancer/treatment; ECOG performance status < 2; stable use of fatigue-relevant meds/supplements allowed if unchanged ≥ 2 weeks	Moderate–severe pain (NRS ≥ 4); pleural effusion/ascites/peripheral edema grade ≥ 3 (CTCAE v4.03); anemia requiring transfusion; hypothyroidism; psychological or mental disorder; nutritional dystrophy; paralytic/atrophic myopathy incl. myasthenia gravis; alcohol or psychotropic drug abuse; pregnant or breastfeeding; planned surgery/chemotherapy/ radiotherapy during study; AST or ALT ≥ 2 × ULN; serum creatinine ≥ 1.5 × ULN	Randomized, double-blind, placebo-controlled preliminary trial; 1:1 allocation with permuted blocks; intention-to-treat analysis with LOCF	Primary: BFI; Secondary: HADS; EORTC QLQ-C30; immunoregulatory tests; safety	Breast, gastrointestinal, lung, head and neck, urogenital; mostly locoregional disease (stage I–III; one stage IV)	ECOG 0–1 (eligibility required < 2)	BFI score change through week 3 (end of treatment) and trajectory to week 6	Sipjeondaebo-tang (SDT) 3 g orally three times daily vs. matched placebo for 3 weeks; both groups received standardized fatigue-relief education	BFI improved in both arms and was significantly better with SDT at week 3 (mean 3.56 ± 1.18 vs. 4.63 ± 1.83); ANCOVA adjusted difference favoring SDT (*p* ≈ 0.04). EORTC QLQ-C30 global health improved more with SDT (*p* = 0.02). HADS and immune markers showed no between-group differences. No significant toxicities (one grade 1 dyspepsia in SDT; one grade 1 pruritus in placebo). Fatigue reduction waned by week 6 in both arms	Korea
Martins et al. [20]	2016	NR	NR	Questionnaires at D1, D21, D42, D63; survival monitored during study period (~30 months)	Adults with localized or locally advanced head and neck cancer (stage I–IV) with indication for concurrent chemoradiotherapy; with/without prior surgery or induction chemotherapy	Chronic diseases causing fatigue (e.g., chronic renal failure, fibromyalgia, chronic anemia, untreated hypothyroidism); oral mucosa problems unrelated to cancer; untreated depression or anxiety	Phase II, randomized, double-blind, placebo-controlled trial	Primary: effect of guaraná on cancer-related fatigue during chemoradiotherapy; Secondary: quality of life (QoL) domains, toxicity (CTCAE), survival	Head and neck squamous cell carcinoma (sites included oral cavity, oropharynx, nasopharynx, larynx)	KPS tracked; % with KPS reduction reported; baseline threshold not specified	FACIT-F (FACT-F) fatigue scale; additional QoL instruments: FACIT-HN, EORTC QLQ-C30, QLQ-H&N35	Guaraná (*Paullinia cupana*) dry extract 50 mg PO twice daily during chemoradiotherapy	No sustained benefit of guaraná on fatigue or QoL vs. placebo; transient early improvements in some H&N35 domains; later greater weight loss and more NG tube/analgesic use in guaraná arm; no significant toxicity differences; no OS difference	Brazil
Miranda et al. [21]	2009	NR	NR	≈5 weeks (start, mid, end of RT course)	Consenting adults with histologically confirmed early-stage breast cancer indicated for adjuvant RT	Prior RT; anemia; clinical depression; inability to consen; contraindications to guaraná (uncontrolled hypertension, prior arrhythmia, insomnia)	Randomized, double-blind, placebo-controlled crossover during adjuvant RT; 36 pts; 75 mg guaraná daily vs. placebo; crossover at mid-RT	Fatigue and depression scores (Chalder Fatigue Scale, MD Anderson Brief Fatigue Inventory, Beck Depression Inventory II) compared across phases	Breast cancer (predominantly ductal), stages I–II	NR	Change in Chalder and BFI scores	Guaraná extract 75 mg PO daily vs. placebo	No statistically significant differences vs. placebo for fatigue or depression; no significant toxicity observed	Brazil
Mirzaei et al. [19]	2022	October 2017–July 2018	2017–2018	4 weeks	Women 18–70 with histologically confirmed breast cancer referred for chemotherapy; developed fatigue after starting chemotherapy and had no prior fatigue	Unstable cardiopulmonary disease; proteinuria; AST > 3 × ULN; disabling lung disease; history of asthma; uncontrolled pain; severe infection; use of drugs that affect fatigue; active treatment for anemia; allergy to honey/saffron/rose components	Randomized, double-blind, placebo-controlled clinical trial (two parallel arms)	Primary: change in fatigue at weeks 0 and 4 via VAFS, FSS, and CFS (total and subscales); Secondary: safety/adverse effects	Breast cancer (patients receiving chemotherapy regimens including anthracycline/cyclophosphamide or taxane ± trastuzumab)	Not reported	VAFS; FSS; CFS (physical, affective, cognitive subscales)	Jollab syrup 20 mL three times daily for 4 weeks (per 100 mL: 79.96 g honey, 20 g rose water, 40 mg saffron extract)	Jollab significantly reduced fatigue vs. placebo: VAFS and FSS decreased (*p* < 0.001), CFS total and physical and cognitive subscales improved; affective subscale not significant; no safety concerns; higher dropout in placebo.	Iran
Ng et al. [18]	2024	2019 (June–August)	2019 (trial period concurrent with enrollment)	9 weeks (baseline, weeks 3, 6, 9)	Adults with solid tumors (stage II–IV) undergoing chemotherapy; prescreened with BFI; included if fatigue increased by ≥1 point between assessments; ITT analyzed	Excluded if BFI fatigue severity did not increase by ≥1 between prescreen and next cycle	Multicenter, randomized, double-blind, placebo-controlled, phase II, parallel assignment	Primary: BFI and VAS-F fatigue scores (baseline, weeks 3, 6, 9); Secondary: SF-36 vitality/other domains; urinary F2-isoprostanes; ECOG status; AEs; labs/vitals	Bone, breast, lung, skin, gynecologic, GI andcolorectal, head and neck, other	ECOG 0–1 at baseline (≈50% ECOG 0; ≈50% ECOG 1 across groups)	BFI and VAS-F; change over time vs. placebo	Nuvastatic™ (C5OSEW5050ESA) 1000 mg orally three times daily for 9 weeks	Significant reductions vs. placebo: BFI (partial η^2^ = 0.333, *p* < 0.001) and VAS-F (partial η^2^ = 0.083, *p* < 0.001). Improved SF-36 (partial η^2^ = 0.243, *p* < 0.001). Urinary F2-isoprostanes decreased (mean diff 55.57; *p* < 0.001). Mild AEs only (vomiting 0.9%, fever 5.4%, headache 2.7%); no severe AEs reported	India
Sette (I) et al. [17]	2018	Not reported	Not reported	21 days after randomization; crossover after 3 weeks with 1-week washout	Women with early breast cancer (stages I–III) starting adjuvant chemotherapy; increased fatigue on BFI after the first cycle	Uncontrolled hypertension; arrhythmias/heart disease; prior serious cardiovascular event; insomnial; depression/psychiatric illness; anemia; chronic renal failure; hypothyroidism; fibromyalgia	Study 1: double-blind randomized cross-over PC-18 37.5 mg BID vs. placebo	Primary: % of patients with ≥1 SD decrease in BFI at 21 days post-randomization; Chalder also assessed	Early breast cancer (stages I–III)	Not reported	BFI and Chalder Fatigue Questionnaire	PC-18 (purified *Paullinia cupana*) 37.5 mg BID	PC-18 not superior to placebo; notable placebo antifatigue effect; no significant toxicities	Brazil
Sette (II) et al. [17]	2018	Not reported	Not reported	21 days after randomization	Women with early breast cancer (stages I–III) starting adjuvant chemotherapy; increased fatigue on BFI or Chalder after the first cycle	Uncontrolled hypertension, arrhythmias/heart disease; prior serious cardiovascular event; insomnia; depression/psychiatric illness; anemia; chronic renal failure; hypothyroidism; fibromyalgia	Study 2: phase II double-blind randomized parallel three-arm (placebo vs. PC-18 7.5 mg BID vs. PC-18 12.5 m BID)	Primary: % of patients with ≥1 SD decrease in BFI at 21 days; Chalder also assessed.	Early breast cancer (stages I–III)	Not reported	BFI and Chalder Fatigue Questionnaire	PC-18 (purified *Paullinia cupana*) 7.5 mg BID or 12.5 mg BID	PC-18 not superior to placebo at either dose; placebo group had significant rise in serum magnesium; multivariate: higher baseline magnesium and BFI and 12.5 mg dose associated with higher post-treatment BFI; no significant toxicities	Brazil
Yennurajalingam et al. [16]	2017	Not reported	Intervention for 28 days; assessments at baseline, days 8/15/29, plus 1 month post-treatment	Primary at day 29; safety also at ~2 months (incl. 1 month post-treatment)	Adults with cancer and CRF ≥ 4/10 on ESAS, present most of the day for ≥2 weeks; normal cognition; ECOG ≤ 2; no uncontrolled pain or depressive symptoms	Infections; uncontrolled diabetes; anticoagulants or systemic steroids; hepatitis A/B/C; significant uncontrolled hypertension or symptomatic tachycardia; active psychiatric illness; current use of ginseng or stimulants	Randomized, double-blind, placebo-controlled parallel-group trial	Primary: change in FACIT-F fatigue subscale fro baseline to day 29; Secondary: QoL (FACIT domains), mood (HADS), ESAS symptoms (incl. fatigue item), Global Symptom Evaluation, 6 min walk test, handgrip strength, safety (CTCAE v4.0)	Advanced solid tumors (various: genitourinary, breast, thoracic, gastrointestinal, others)	ECOG 0–2	FACIT-F fatigue subscale	*Panax ginseng* extract 400 mg twice daily vs. matching placebo for 28 days; oral administration	Both groups improved on fatigue; PG not superior to placebo at day 29 (ΔFACIT-F 7.5 vs. 6.5; *p* = 0.67). Fewer any-grade AEs and fewer grade ≥3 AEs with PG (3–5: 1/63 vs. 9/64)	USA
Chan et al. [26]	2025	Unclear, looks like 2019–2022	2019–2022	10 weeks	≥21 years old; reported a fatigue screen score of ≥4 in the past 7 days; had completed surgery, chemotherapy, or RT and were not planned to receive adjuvant therapy during the study period, except for aromatase inhibitors or ovarian suppression for breast cancer survivors	Metastases, cancer recurrence; untreated fatigue-causing co-morbidities; on fatigue-inducing medications; taking warfarin; receiving or planning to receive TCM treatment; breastfeeding or intending to conceive	RCT	QoL, CRF, perceived cognition, BDNF levels, BDNF genotyping	Breast, lymphoma, endometrial, pancreatic, ovarian, lung, uterine	ECOG 0–15 (100.0)/11 (84.6); 1–0 (0.0)/2 (15.4)	MFSI-SF	XBYRT was administered as oral granules at a dose of 24 g once daily, dissolved in hot water, for 8 weeks	No significant difference found in QoL; I—improved emotional fatigue at T3 and higher BDNF levels at T2 and T3; I—better perceived cognitive impairment at T2 and T3, and overall perceived cognitive function at T3.	Singapore
Chen et al. [25]	2012	NR	NR	8 weeks	Advanced cancer with a fatigue score of at least 4 and a life expectancy of at least three months	Pregnancy or breastfeeding; uncontrolled systemic diseases; use of any central nervous system stimulators or standard cancer chemotherapy within the previous 30 days; KPS < 30%	RCT	CRF, AE	Breast, gynecologic, GI, head and neck, respiratory, male reproductive, others	Median KPS I 70/C 70	FIRR	PG2 was administered as an intravenous infusion of 500 mg, three times weekly, over 4 weeks; during the second cycle, all patients received PG2 for an additional 4 weeks	PG2 improved CRF, with higher fatigue-improvement response at week 1 vs. placebo (57% vs. 32%; *p* = 0.043). At week 4, 60% achieved ≥10% BFI-T reduction; 72% of responders had ≥20% improvement. Benefits were sustained in 82%, and 71% of initial non-responders improved after prolonged treatment	Taiwan
Del Giglio et al. [24]	2013	NR	NR	3 weeks	Adults with cancer at any stage receiving systemic chemotherapy	History of hypothyroidism; psychiatric disorder; anemia; prior antineoplastic treatment; insomnia; angina; uncontrolled hypertension; neurologic disorders; cardiovascular disease	Initially uncontrolled, open study; then, RCT	Fatigue, Anxiety and Depression, Sleep Quality Questionnaire, AEs	Breast, colorectal, lung/pleura, head and neck, ovarian, bone, stomach, urethra, pancreas, prostate, biliary tract	NR	FACIT-F; Chalder Fatigue Scale	PC-18 was administered orally at a dose of 37.5 mg twice daily for 21 days	Compared with baseline, BFI scores improved significantly after 3 weeks of PC-18 (mean reduction 2.5 points; *p* = 0.0002)	Brazil
Barton et al. [29]	2010	2005–2006	2005–2006	8 weeks	Adults with cancer-related fatigue ≥ 4/10 for ≥1 month; life expectancy ≥ 6 months; various solid tumors	Other medical causes of fatigue ruled out	Multicenter, randomized, double-blind, placebo-controlled pilot trial	Primary: BFI activity-interference AUC; Secondary: BFI usual fatigue, SF-36 vitality, PSQI, quality-of-life domains	Mixed solid tumors (breast, colon, lung, others)	Not reported.	BFI); SF-36 vitality	American ginseng 750–2000 mg/day	High-dose ginseng showed trend toward improvement, but nonetheless non-significant	USA
Barton et al. [28]	2013	2008–2011	2008–2011	8 weeks	Adults with cancer-related fatigue ≥ 4/10 for ≥1 month, diagnosed within past 2 years; on or recently completed chemotherapy/RT	Brain/CNS lymphoma; changes in treatment planned; alternative causes of fatigue; pain/insomnia ≥ 4/10; steroid/opioid use; prior/current ginseng use	Randomized, double-blind, placebo-controlled phase III trial	Primary: MFSI-SF general fatigue at 4 weeks; Secondary: fatigue at 8 wks, POMS, BFI, toxicity	Mixed solid and hematologic cancers (predominantly breast)	Not reported	MFSI-SF general fatigue; POMS; BFI	American ginseng 2000 mg/day	No significant difference at 4 weeks (mean MFSI-SF change +14.4 vs. +8.2; *p* = 0.07); significant improvement at 8 weeks, with greater fatigue reduction vs. placebo (+20.0 vs. +10.3 points, *p* = 0.003), most pronounced in patients receiving active cancer therapy	USA
Campos et al. [27]	2011	2008–2009	2008–2009 (3-cycle intervention)	~49 days total	Women 22–70 with histologically confirmed breast cancer starting first chemotherapy cycle; fatigue worsened ≥1 category after first cycle; provided informed consent	Hypothyroidism; depression/psychiatric disease; anemia; prior antineoplastic therapy; insomnia; cardiovascular disease; uncontrolled hypertension; neurologic disorders; use of antidepressants/anxiolytics/sleep aids; severe fatigue at baseline or failure to worsen after cycle 1	Phase II, randomized, double-blind, placebo-controlled crossover trial (21 days per arm, 7-day washout)	Primary: FACIT-F global fatigue score; Secondary: FACT-ES, BFI, Chalder Scale, PSQI, HADS, safety	Breast cancer (all stages)	Not reported.	FACIT-F improvement; BFI and Chalder changes	Guaraná extract 50 mg twice daily	Guaraná vs. placebo: FACIT-F increased by +14.2 points at day 21 and +23.5 points at day 49 (vs declines with placebo; *p* < 0.01); ≥1-SD FACIT-F improvement in 52–66% vs. 10–13% with placebo. BFI decreased by −3.2 points (day 21) and −2.2 points (day 49) vs. minimal/no improvement with placebo (*p* < 0.01). Chalder Fatigue score decreased by −4.6 points at day 21 (*p* < 0.01)	Brazil

Table 1: Features of included studies: AEs: Adverse Events; ALT: Alanine Aminotransferase; ANCOVA: Analysis of Covariance; AST: Aspartate Aminotransferase; AUC: Area Under the Curve; BDNF: Brain-Derived Neurotrophic Factor; BFI: Brief Fatigue Inventory; BID: Twice Daily; CFS: Chalder Fatigue Scale; CNS: Central Nervous System; CRF: Cancer-Related Fatigue; CTCAE: Common Terminology Criteria for Adverse Events; DFS: Disease-Free Survival; ECOG: Eastern Cooperative Oncology Group Performance Status; EORTC QLQ-C30: European Organization for Research and Treatment of Cancer Quality of Life Questionnaire Core 30; ESAS: Edmonton Symptom Assessment System; FACT-ES: Functional Assessment of Cancer Therapy–Endocrine Symptoms; FACT-F: Functional Assessment of Cancer Therapy–Fatigue; FACIT-F: Functional Assessment of Chronic Illness Therapy–Fatigue; FACIT-HN: Functional Assessment of Chronic Illness Therapy–Head and Neck; FIRR: Fatigue Interference Rate Reduction; FSS: Fatigue Severity Scale; GI: Gastrointestinal; GM-CSF: Granulocyte-Macrophage Colony-Stimulating Factor; HADS: Hospital Anxiety and Depression Scale; ITT: Intention-to-Treat; KPS: Karnofsky Performance Status; LOCF: Last Observation Carried Forward; MD: MD Anderson Identifier; MFSI-SF: Multidimensional Fatigue Symptom Inventory–Short Form; NAC: Neoadjuvant Chemotherapy; NG: Nasogastric; NR: Not Reported; NRS: Numerical Rating Scale; OS: Overall Survival; PC-18: Purified *Paullinia cupana* Extract/Pharmaton Complex 18; PG: *Panax Ginseng*; PG2: Polysaccharide Peptide Ginseng Extract; pCR: Pathological Complete Response; PO: Per os (By Mouth); POMS: Profile of Mood States; PSQI: Pittsburgh Sleep Quality Index; pts: Patients; QoL: Quality of Life; QLQ-H&N35: EORTC Quality of Life Questionnaire–Head and Neck 35; RCT: Randomized Controlled Trial; RT: Radiotherapy; SD: Standard Deviation; SDT: Sipjeondaebo-tang; SF-36: Short Form-36 Health Survey; SSRIs: Selective Serotonin Reuptake Inhibitors; TCM: Traditional Chinese Medicine; T2: Timepoint 2; T3: Timepoint 3; ULN: Upper Limit of Normal; VAS-F: Visual Analog Scale for Fatigue; VAFS: Visual Analog Fatigue Scale; η^2^: Eta-Squared.

**Table 2:** This table summarizes the methodological quality of the 14 randomized controlled trials included in the meta-analysis. Each study was evaluated across five domains of the Cochrane Risk of Bias 2.0 tool: randomization process, deviations from intended interventions, missing outcome data, measurement of the outcome, and selection of the reported result. Overall risk of bias was determined based on domain-level judgments.

RoB Category	Trials (*n*)	Studies
Low Risk	2	Barton et al. [28]; Yennurajalingam et al. [16]
Some Concerns	6	Barton et al. [29]; Campos et al. [27]; Mirzaei et al. [19]; Ng et al. [18]; Chen et al. [25]; Lee et al. [22]
High Risk	6	del Giglio et al. [24]; Guglielmo et al. [23]; Miranda et al. [21]; Chan et al. [26]; Sette et al. [17]; Martins et al. [20]

**Table 3:** Subgroup analysis of overall effects according to herbal class, risk of bias, disease timepoints, and treatment duration.

Subgroup/Category	Representative Trials	k (Trials)	Pooled SMD	95% CI	I^2^ (%)	Interpretation
Herbal Class						
Ginseng (*Panax* spp.)	Barton et al. [28]; Barton et al. [29]; Yennurajalingam et al. [16]; Guglielmo et al. [23]	4	0.22	−0.11 to 0.53	12.1	Not statistically significant
Guaraná (*Paullinia cupana*)	Campos et al. [27]; del Giglio et al. [24]; Miranda et al. [21]; Sette et al. [17]; Martins et al. [20]	5	0.15	−0.46 to 0.76	71.4	Very imprecise; not statistically significant; wide range from possible harm to benefit; highly heterogeneous
Other herbal formulations (Jollab, Nuvastatic, PG2, XBYRT, SDT)	Mirzaei et al. [19]; Ng et al. [18]; Chan et al. [26]; Chen et al. [25]; Lee et al. [22]	5	0.62	0.33 to 0.91	0	Statistically significant; no heterogeneity
Methodological Quality						
Low risk of bias	Barton et al. [28]; Yennurajalingam et al. [16]	2	0.34	−3.31 to 3.99	64.0	Non-statistically significant trend favoring phytotherapy; very imprecise
Some concerns	Barton et al. [29]; Campos et al. [27]; Mirzaei et al. [19]; Ng et al. [18]; Chen et al. [25]; Lee et al. [22]	5	0.56	0.25 to 0.87	34.2	Moderate effect; statistically significant; driven by small studies
High risk of bias	Del Giglio et al. [24]; Guglielmo et al. [23]; Miranda et al. [20]; Chan et al. [26]; Sette et al. [17]; Martins et al. [20]	6	−0.01	−0.43 to 0.42	32.8	Non-significant; no clear trend
Disease Timepoint						
Advanced disease	Chen et al. [25]; Martins et al. [20]; Yennurajalingam et al. [16]	3	0.08	−0.89 to 1.04	60.2	Non-statistically significant
Early disease	Lee et al. [22]; Sette et al. [17]; Miranda et al. [21]	3	0.24	−0.83 to 1.30	61.7	Non-significant; trend toward benefit
Survivorship	Chan et al. [26]; Barton et al. [28]; Guglielmo et al. [23]	3	0.31	−0.88 to 1.51	50.4	Non-significant; trend toward benefit
Treatment Duration						
<4 weeks	Del Giglio et al. [24]; Lee et al. [22]; Miranda et al. [21]; Mirzaei et al. [19]; Sette et al. [17]; Yennurajalingam et al. [16]	6	0.16	−0.13 to 0.46	12.3	Non-statistically significant
≥4 weeks	Barton et al. [28]; Barton et al. [29]; Chen et al. [25]; Chan et al. [26]; Guglielmo et al. [23]; Martins et al. [20]; Ng et al. [18]	7	0.31	−0.11 to 0.72	65,9	Non-significant; trend toward benefit

## Data Availability

All data was obtained from publicly available articles.

## Supplementary Materials

The following supporting information can be downloaded at: https://www.mdpi.com/article/10.3390/diseases14020039/s1; Table S1: PRISMA checklist.
